# Alternate primers for whole-genome SARS-CoV-2 sequencing

**DOI:** 10.1093/ve/veab006

**Published:** 2021-02-04

**Authors:** Matthew Cotten, Dan Lule Bugembe, Pontiano Kaleebu, My V.T. Phan

**Affiliations:** 1 MRC/UVRI & London School of Hygiene and Tropical Medicine, 51-59 Nakiwoggo Road, Entebbe, Uganda; 2 UK Medical Research Council–University of Glasgow Centre for Virus Research, Glasgow, UK; 3 Uganda Virus Research Institute, Entebbe, Uganda

**Keywords:** SARS-CoV-2, COVID-19, primers, next generation sequencing

## Abstract

As the world is struggling to control the novel Severe Acute Respiratory Syndrome Coronavirus 2 (SARS-CoV-2), there is an urgency to develop effective control measures. Essential information is encoded in the virus genome sequence with accurate and complete SARS-CoV-2 sequences essential for tracking the movement and evolution of the virus and for guiding efforts to develop vaccines and antiviral drugs. While there is unprecedented SARS-CoV-2 sequencing efforts globally, approximately 19 to 43 per cent of the genomes generated monthly are gapped, reducing their information content. The current study documents the genome gap frequencies and their positions in the currently available data and provides an alternative primer set and a sequencing scheme to help improve the quality and coverage of the genomes.

## 1. Introduction

Since the first report on 30 December 2019 in Wuhan China and the WHO declaration of the pandemic on 12 March 2020, the novel Severe Acute Respiratory Syndrome Coronavirus 2 (SARS-CoV-2) (Holmes and Zhang 2020) and the associated disease Coronavirus Disease 2019 (COVID-19) (Li et al. 2020; Yang et al. 2020) have continued to spread throughout the world, causing >46 million infections and >1,200,000 death globally (Gardner et al. 2020). The virus genome sequences carry important information, which can be used to interpret the virus transmission, evolution patterns and origin tracing. Furthermore, accurate and complete genomic sequences are essential for monitoring diagnostics and developing novel therapeutics and vaccines. We have seen an unprecedented amount of virus sequencing with over 130,000 complete or nearly complete genome sequences of SARS-CoV-2 now available in the GISAID database by the end of September 2020 (Shu and McCauley 2017). Most of the sequences have been generated by next generation sequencing using targeted amplicon methods. A scan through SARS-CoV-2 genomes from GISAID with the filter ‘complete genome’ revealed a high frequency of gaps occurring across the genome, influencing the overall genomics quality and interpretation. Here, we describe an alternate primer scheme for whole-genome sequencing to improve the genome sequence quality and coverage.

## 2. Documenting the problem

**Figure 1.**
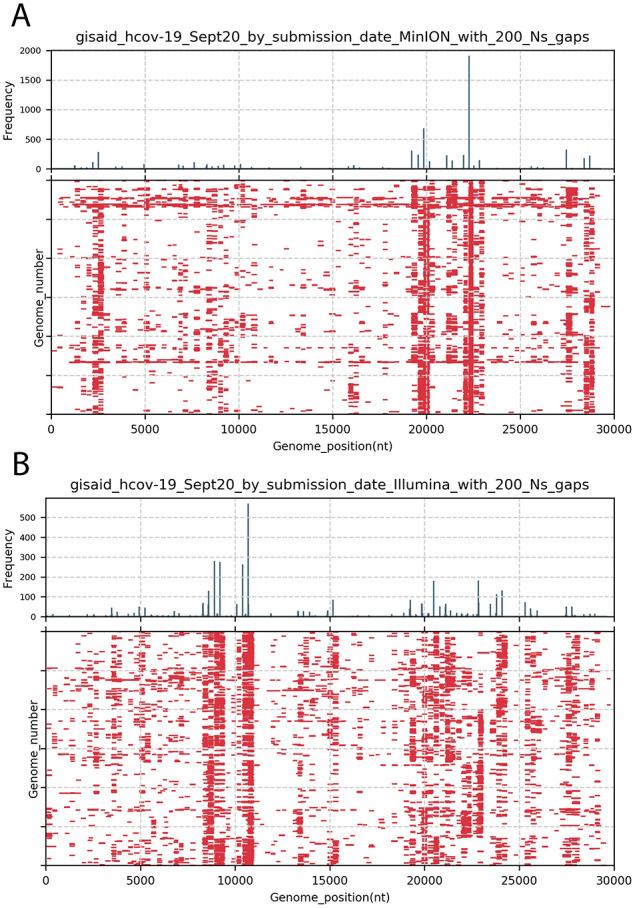
(updated graphics) Positions of 200 nt gaps across SARS-CoV-2 genomes listed as complete in GISAID. Genomes deposited in September 2020 (n = 38,228) were retrieved from GISAID, sorted by sequencing platform (MinION versus Illumina) and genomes with at least one instance of 200 N were collected. Panel A presents gaps in the first 3,000 MinION-generated genome sequences deposited that contained at least on 200 N motif. Gaps >= 200 nt in each genome are indicated with red bars. The upper panel histogram shows the frequency (in 30 nt bins) of gaps >=200 nt motifs by start position on genomes. Panel B is the same analysis of the first 3,000 Illumina-generated genome sequences in September 2020 that contained at least one 200 N motif.

We retrieved genomes deposited to GISAID in September 2020 (9 months into the pandemic), using the ‘complete genome’ filter and sorting the genomes by sequencing platforms information included in the metadata. Figure 1 illustrated the positions across the 30 kb genome of every stretch of 200 Ns (N200; nearly the size of an amplicon) in the first genomes deposited in September 2020 using the Illumina platform (Panel A; N = 3,000) versus MinION platform (Panel B; N = 3,000). Histograms of gap frequencies across the genomes are shown for each platform. The gaps are not randomly distributed but occur with higher frequency in a subset of positions across the genome. Although genomes generated by the two platforms (Illumina and MinION) show similar problem regions (nt 8,000–11,000, and nt 19,000–24,000 relative to the reference genome NC_045512), the patterns are not completely identical. Given the use of several primer amplification schemes, we suspect the gaps in coverage may be due to unexpected primer interactions, complicated sequence regions (odd composition or secondary structure), issues with primer trimming during quality control of read data, or some combinations of these factors.

The phenomenon is unlikely to be due to an isolated set of genomes as we observed similar N200 frequencies in genomes submitted from each month of the pandemic (Table 1), suggesting that gaps in coverage is a more general phenomenon. Of note, genomes generated using Ion Torrent show much lower levels of N200 (Table 1). The very low frequency of large gaps in the Ion Torrent data may be due to the use of a dedicated alternative primer set (Alessandrini et al. 2020). There have been discussions and reports on the SARS-CoV-2 genome changes due to sequencing errors as well as long gaps in the genomes due to missing amplicons from the amplicon-approach sequencing (De Maio et al. 2020; Page et al. 2020). Updates of the ARTIC primers have been presented in late March 2020 to address these issues (Quick and Loman 2020; Tyson et al. 2020). Additional reports of longer amplicon methods have been published (Eden et al. 2020; Freed et al. 2020; Gonzalez-Reiche et al. 2020), including methods to use a subset of ARTIC primers to generate longer amplicons (Itokawa et al. 2020).

**Table 1.** Frequency of SARS-CoV-2 genomes with 1 or more 200 nt gaps (N200) by month and by sequencing platform.

Deposition period	Complete genomes^a^	Genomes with 1 or more N200^b^	%. with 1 or more N200^c^	Illumina total^d^	Illumina % with N200^e^	MinION total^f^	MinION % with N200^g^	Ion Torrent total^h^	Ion Torrent % with N200^i^	Method unclear^j^	Method unclear % with N200^k^
1–31 January 2020	54	1	2	9	0	2	0	0	0	43	2
1–29 February 2020	126	2	2	44	0	9	0	5	0	65	4
1–31 March 2020	2,872	559	19	1,518	16	548	32	35	6	771	18
1–30 April 2020	12,411	3,745	30	4,970	38	1,286	27	264	0	5,424	26
1–31 May 2020	19,787	8,606	43	8,634	52	2,634	30	529	0	7,990	42
1–30 June 2020	21,665	8,723	40	7,043	36	3,844	35	629	2	10,149	47
1–31 July 2020	17,986	4,834	27	4,965	23	1,585	33	471	2	10,965	29
1–31 August 2020	17,276	4005	23	11,074	22	2,270	26	486	0	3,446	28
1–30 September 2020	38,227	10,611	28	22,740	23	7,973	44	580	1	6,934	28

a Number of genomes with the annotation ‘complete’ retrieved from GISAID (https://www.gisaid.org/).

b Genomes were sorted by the presence or absence of the sequence N200.

c ((The number of genomes with at least one N200)/total number of genomes)*100.

d Number of genomes in GISAID for this period generated using any of the Illumina methods as noted in the GISAID ‘Sequencing technology metadata’.

e ((The number of Illumina genomes with at least one N200)/total number of genomes)*100.

f,g Number of genomes in GISAID for this period generated using any of the MinION methods as noted in the GISAID ‘Sequencing technology metadata’ and their percentage.

h,i Number of genomes in GISAID for this period generated using any of the Ion Torrent methods as noted in the GISAID ‘Sequencing technology metadata’ and their percentage.

j,k Number of genomes in GISAID for this period generated using unclear methods as noted in the GISAID ‘Sequencing technology metadata’ and their percentage.

However, the percentage of reported complete genomes in GISAID with 1 or more N200s continues, with 10,611 (28 per cent) of the 38,228 genomes deposited in September 2020 having 1 or more N200 gaps (Table 1), indicating the challenges remain largely unsolved.

## 3. Detailed analysis of gaps

**Figure 2.**
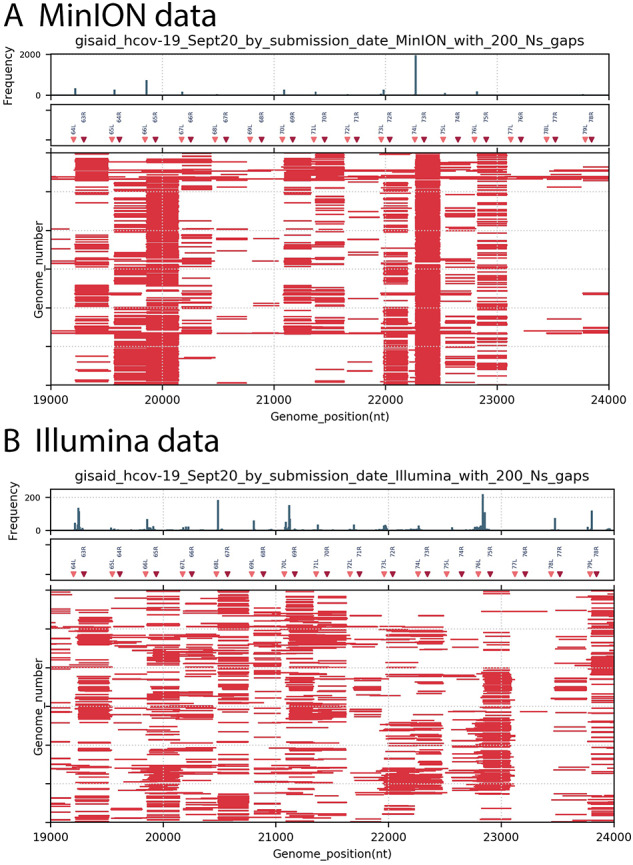
Positions of 200 nt gaps across SARS-CoV-2 genomes stratified by MinION or Illumina, in region nt 19,000 to 24,000. Genomes deposited in September 2020 as ‘complete’ were retrieved from GISAID, sorted by sequencing platform and by the presence of at least one N200 motif. For clarity, only the first 3,000 genomes in each set were plotted. Similar to Figure 1, gaps >= 200 nt in each genome are indicated with red bars. The upper panel histogram shows the frequency (in 30 nt bins) of gaps >= 200 nt motifs by start position on genome, the middle panel plots the positions of ARTIC v.1 primers in the region (pink = forward ‘left’ primers, red = reverse ‘right’ primers). Panel A: MinION-derived genome sequences, Panel B: Illumina-derived genome sequences.

A more focused analysis of the frequent gaps is provided in Fig. 2. The gap pattern between nt 19,000 and 24,000 (relative to the reference genome NC_045512) is shown for both MinION and Illumina sequences (first 3,000 genomes of each deposited in September 2020 with at least one 200 N motif). For reference, the positions of the ARTIC primers (v.1) in the region are indicated (middle panel). A histogram of gap start positions (top panel) and the individual genome gaps (bottom panel) are also shown.

The peaks of gap start positions frequently lie between forward primerL from Amplicon n and reverse primerR from Amplicon n−1, for both MinION and Illumina data. Because of overlapping amplicons commonly used, if a single amplicon is missing from the sequencing library (amplicon 74 for example), the resulting gap in coverage would not be the complete amplicon 74 but would span from the 3′ end of the adjacent amplicon 73 (after primer and quality trimming) to the 5′ end of adjacent amplicon 75 (after primer and quality trimming). The calculated gaps generated by such amplicon loss have a median length of 270.5 nt, which is close to the the observed median gap length in the MinION data (258 nt) or Illumina data (262 nt) from September 2020. This arrangement is outlined in the Supplementary Fig. S1, Panel A.

## 4. Alternate primers as a potential solution to avoid gapped genomes

We explored an alternate set of amplification primers (termed the **Entebbe primers**) designed using methods we had previously used for MERS-CoV (Cotten et al. 2013), Norovirus (Cotten et al. 2014), RSV (Agoti et al. 2015) and Yellow Fever virus (Phan et al. 2019). Important for the design were the amplicon size and the primer placement. For their implementation, the use of primers for the reverse transcription step, and the multiplexing of the amplicons in two staggered sets were important for the PCR. Our experience had suggested an optimum amplicon size of around 1500 bp. The larger amplicons reduced the total primers content of the reactions but still allowed high reverse transcription efficiency (which, in our hands, declined beyond 1500 nt). Here we describe primers designed for whole-genome sequencing of SARS-CoV-2, as well as sharing the detailed laboratory methods that we used for reverse transcription, PCR amplification and MinION library preparation to successfully sequence the SARS-CoV-2 genome.

**Figure 3.**
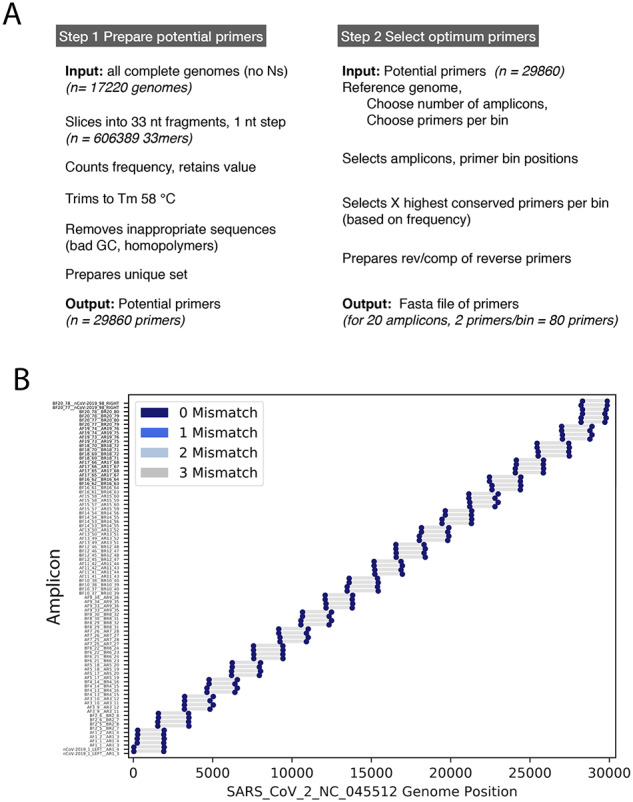
Primer design and amplicon layout. Panel A: The two main steps involved in primers generation and selection are shown. Panel B: The layout of the twenty amplicons across the SARS-CoV-2 genome is shown in lower panel. The blue markers indicate target positions in the SARS-CoV-2 genome (NC_045512 used here), the grey bars indicate the resulting amplicon.

Briefly, the primer design (Fig. 3A) started with the set of complete SARS-CoV-2 genome sequences available in the GISAID database on 22 June 2020 (N = 21,687). Spaces and disruptive characters were removed from the sequence IDs and the sequences were further screened to remove genomes containing gaps of 6Ns or more, resulting in 17,220 clean genome sequences. Next, all sequences were sliced into 33 nt strings (33mers), with a 1 nt step and 606,389 unique 33mers were generated. The frequency of each 33mer was counted to identify highly conserved 33mers. This counting method avoids the multiple sequence alignment step commonly used in primer design and becomes prohibitive with large and or diverse genome sets. This alignment-free approach allowed us to use all suitable genome sequences of interest rather than a set that could be conveniently aligned. Finally, primer-like 33mers sequences were generated by trimming the sequences to a calculated desired melting temperature and removing any primers greater than 26 nt.

In the second step, we defined forward and reverse primer target regions (bins) for the amplicons. For SARS-CoV-2, we selected twenty amplicons with an overlap of 300 nt, regularly spaced across the SARS-CoV-2 genome sequence (Fig. 3B). We then selected the top conserved primer sequences (the highest frequency primers) mapping in the 5′ or 3′ 185 nt of each amplicon. For security, the two highest frequency primers per bin were selected for the SARS-CoV-2 sequence, this provided some insurance against primer failure either due to target evolution or unexpected secondary structure. The binning and primer target locations for the final set of primers are shown in Fig. 2 and the final calculated amplicon lengths were 1,495–2,093 nt.

The reverse transcription, PCR amplification and library protocols were modified to accommodate the new primers. Important changes to note are the following. Reverse transcription was performed using the reverse primers and reverse transcription at 42°C. The PCR cycling conditions (using Phusion enzyme) were adjusted for the new T_m_s and an increased elongation time required for the longer PCR products. Finally, the library purification steps were adjusted to recover longer PCR and library products. A detailed step-by-step protocol is provided in the Supplementary material.

## 5. Testing the performance of primers to sequence SARS-CoV-2 using MinION

**Figure 4.**
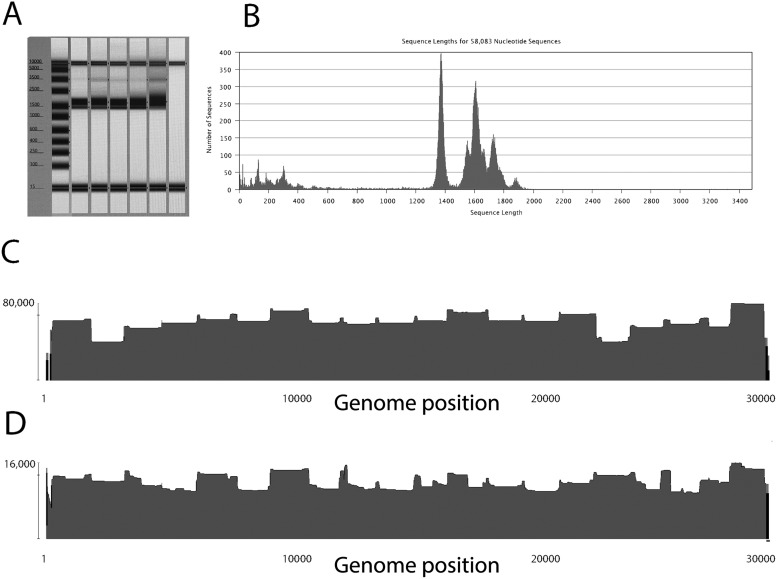
Testing the primer performance. Panel A: PCR product size after pooling of reaction A and B. Expected sizes of amplicons are from 1,500 bp to 2,093 bp before primer trimming. Panel B: MinION reads after quality control, primer, adapter trimming. Panel C: Reads mapped to SARS-CoV-2 reference genome, before amplicon 2 and 16 primer boosting. Panel D: Reads mapped to SARS-CoV-2 reference genome, after amplicon 2 and 16 primer boosting.

We tested the Entebbe primers performance for sequencing SARS-CoV-2 from nucleic acid extracted from positive samples. The amplicon sizes and genome coverage are summarised in Fig. 4. In particular, panels A and B (Fig. 4) illustrate the amplicon products after the reverse transcription and PCR amplification with the expected sizes of 1,400–2,000 bp. These amplicons were then pooled and used for library preparation using the MinION sequencing kits SQK-LSK109. Final libraries were quantified and sequenced using a MinION Flow Cell (R9.4.1). The resulting read data, after quality and primer and adapter trimming, were then mapped to the SARS-CoV-2 Wuhan1 reference genome NC_045512 (Fig. 4, panels C and D) to document sequence coverage across the genome. The twenty individual amplicons are detected in the coverage pattern with small peaks appearing where amplicons overlap. The coverage is consistent across the genome with no missing amplicons and the data were readily assembled into good coverage full genomes. Initial experiments showed that amplicons 2 (spanning nt 2,400) and 16 (spanning nt 23,500) had reduced yields (Fig. 4, panel C). The primer mixes were subsequently adjusted to increase concentrations of amplicon 1 and 16 primers for reverse transcription and PCR (see detailed protocol in Supplementary materials), this improved yields relative to the other amplicons (Fig. 4, panel D).

**Figure 5.**
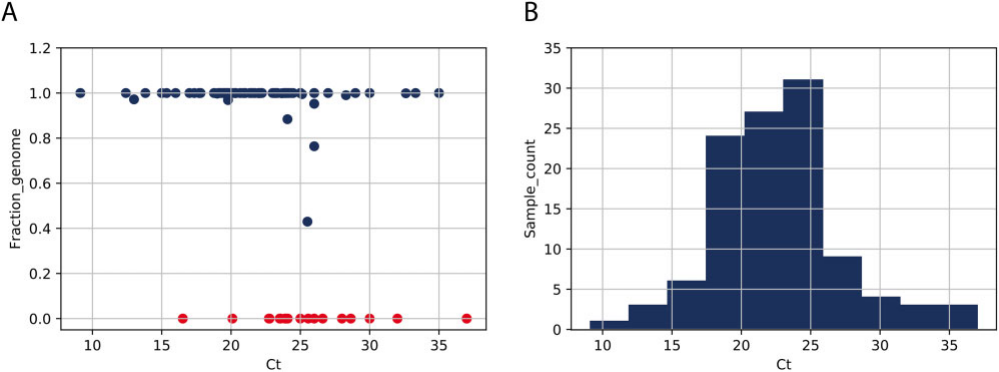
Validation of Entebbe primers. Panel A plots the genome yield (fraction of complete genome) as a function of sample Ct. Fraction genome was calculated by number of nonN nucleotides/29,303 (the length, in nt, of NC_045512 reference genome). Each marker represents a sample, red markers indicate 19 samples that failed to yield sufficient DNA for library, 93 that proceeded to library preparation and sequencing (dark blue markers). Panel B is a histogram of the distribution of the 118 sample Cts.

A set of SARS-CoV-2 clinical samples were tested with the Entebbe primers/protocol. Respiratory swab samples from 111 PCR confirmed cases of SARS-CoV-2 infection were processed for reverse transcription/PCR using the Entebbe primers and protocol as described in Supplementary materials. If sufficient amplicon DNA was generated after PCR, MinION libraries were prepared, samples were sequenced on the MinION flowcells and the resulting data were assembled into genomes. Figure 5A shows the results of this validation test. Complete genomes (fraction genome = 1) were obtained from samples up to Ct 35; nineteen samples failed to yield sufficient amplicon DNA after PCR stage (Fig. 5A, red markers) and five samples yielded genomes with gaps > 842 nt. The PCR failures and the gapped genomes were not strictly associated with higher Cts and their distribution pattern is similar to the overall Ct distribution pattern across the set of samples (Fig. 5, panel B) suggesting other factors such as sample quality, extraction method, storage, might be more critical than Ct in determining sequencing success, at least in samples within the Ct range tested (up to Ct 37).

## 6. Conclusions

Given the urgency of controlling the SARS-CoV-2 pandemic and the importance of having good quality SARS-CoV-2 genomes, we are providing these alternative primers (the Entebbe primers) with detailed step-by-step laboratory protocols to the community with the hope that they benefit from the new design. The costs and efforts of sequencing SARS-CoV-2 in the large case numbers that are currently being seen are substantial and if these new primers result in a higher proportion of gap-less genomes, this will provide added value and will increase the utility of the resulting data.

## Supplementary data

Supplementary data are available at *Virus Evolution* online.

## Supplementary Material

veab006_Supplementary_Data
