# Supplementary material for: Alternate primers for whole-genome SARS-CoV-2 sequencing
Source: Virus Evol. 2021 Feb 4;7(1):veab006. doi: 10.1093/ve/veab006 (PMC7928614; doi:10.1093/ve/veab006)
Supplement: veab006_Supplementary_Data [file veab006_supplementary_data.zip › Supplementary_Material_Figure_1.pdf]

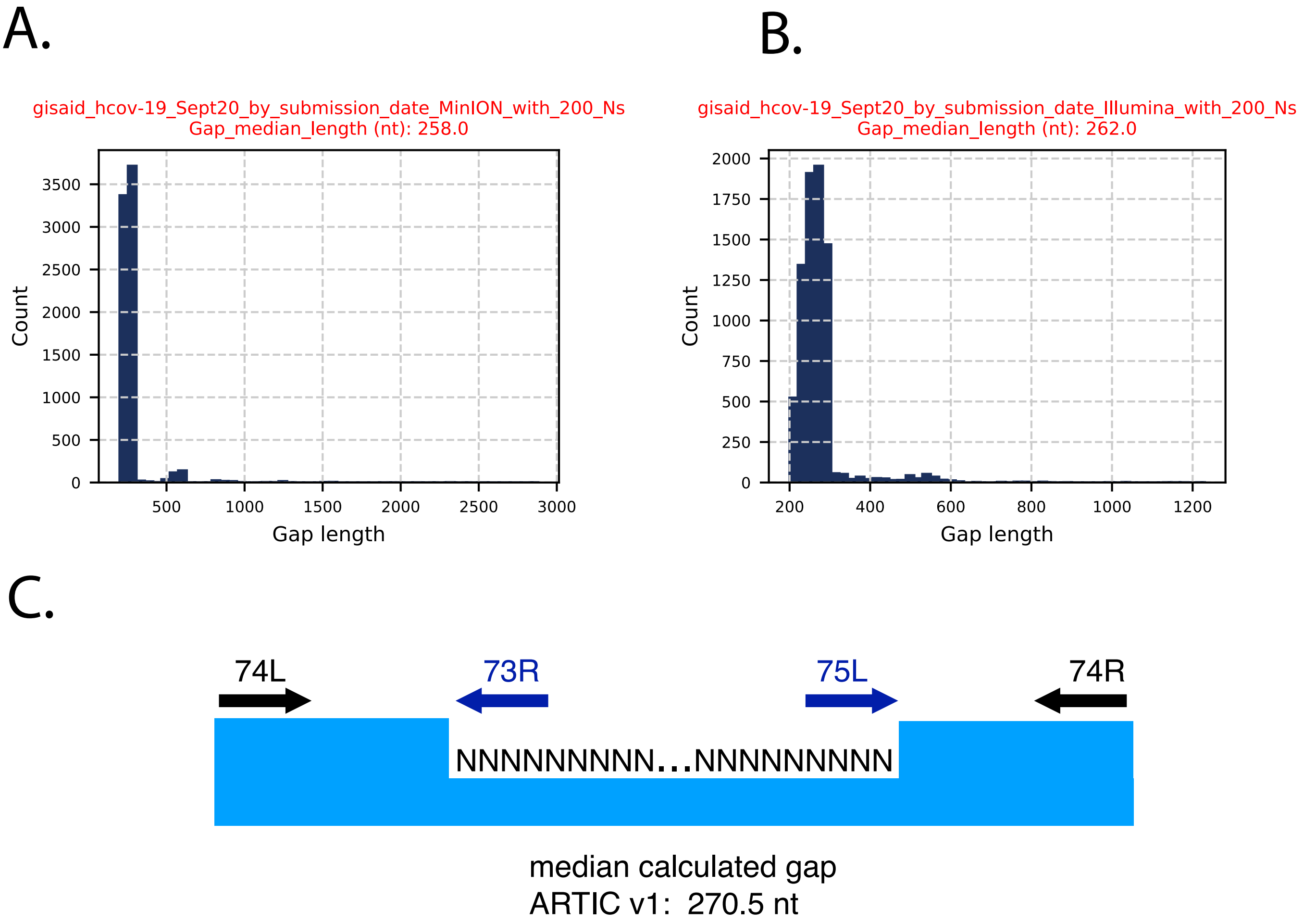
A.
B.
gisaid_hcov-19_Sept20_by_submission_date_MinION_with_200_Ns
Gap_median_length (nt): 258.0
Count
Gap length
gisaid_hcov-19_Sept20_by_submission_date_Illumina_with_200_Ns
Gap_median_length (nt): 262.0
Count
Gap length
C.
74L
73R
75L
74R
NNNNNNNNN...NNNNNNNNN
median calculated gap
ARTIC v1: 270.5 nt
