# Supplementary material for: Alternate primers for whole-genome SARS-CoV-2 sequencing
Source: Virus Evol. 2021 Feb 4;7(1):veab006. doi: 10.1093/ve/veab006 (PMC7928614; doi:10.1093/ve/veab006)
Supplement: veab006_Supplementary_Data [file veab006_supplementary_data.zip › Supplementary_Material.docx]

**Supplementary Materials: 1. Supplementary Material Figure 1, 2. Detailed protocol, 3. Primer table.**

Supplementary Material Figure 1. As described in Figure 1, SARS-CoV-2 genomes deposited in September 2020 (n= 38,228) were retrieved from GISAID, sorted by sequencing platform and the presence of 200N motifs was monitored. Panel A presents a histogram of gap lengths present in the first 3000 MinION-generated genome sequences that contained at least one 200N motif. The median length for all gaps was 258 nt. Panel B present the same analysis for Illumina-generated genome sequences. Panel C presents a typical gap. Because of overlapping amplicons, if a single amplicon is missing (amplicon 74 for example) from the sequencing library, the resulting gap in coverage would not be the complete amplicon 74 but would span from the 3' end of the adjacent amplicon 73 (after primer and quality trimming) to the 5' end of adjacent amplicon 75 (after primer and quality trimming). The calculated gaps generated by such amplicon loss have a median length of 270.5 nt, which is close to the the observed median gap length in the MinION data (258 nt) or Illumina data (262 nt) from September 2020.

**2. Detailed protocol**

We acknowledge the Oxford Nanopore Technologies and the ARTIC Network for their extensive protocols which provided important background details for the method presented here (1) (2). Potential users are strongly advised to study the original protocols carefully. The protocol presented here assumes the users has all the ARTIC and ONT background experience.

**Detailed protocol for reverse transcription, PCR and sequencing library preparation.**

**Practical comments.** Primers were ordered and delivered dry and were dissolved at 100 µM (= 100 pmol/µl) in PCR quality water. Dry shipped primers are reported to have better stability at room temperature (3). Ideal the primers were allowed to dissolve in PCR grade water for at least 3 hours at room temperature or overnight at 4 °C before diluting for use (see below).

**Prepare the following primer mixes:**

**RPM_A**: 21 primers. 3 µl of 100 µM stock of the following Reverse primers plus PCR grade water to 200 µl (= 300 pmol x 21 = 6300 pmol/200 µl = 32 pmol/µl. 2 µl per 20 µl RT reaction = 3.2 pmol/µl = 3.2 µM concentration in reaction) .

**Primers in RPM_A**: nCoV-2019_1_LEFT, AR1_3, AR1_4, AR3_11, AR3_12, AR5_19, AR5_20, AR7_27, AR7_28, AR9_35, AR9_36, AR11_43, AR11_44, AR13_51, AR13_52, AR15_59, AR15_60, AR17_67, AR17_68, AR19_75, AR19_76.

**RPM_B**: 21 primers. 3 µl of 100 µM stock of the following Reverse primers plus PCR grade water to 200 µl. (= 300 pmol x 21 = 6300 pmol/200 µl = 32 pmol/µl. 2 µl per 20 µl RT reaction = 3.2 pmol/µl = 3.2 µM concentration in reaction). **Primer Boost:** For Primers BR2_7, BR2_8, BR16_63, BR16_64 use 9 µl of 100 µM stock.

**Primers in RPM_B:** BR2_7, BR2_8, BR4_15, BR4_16, BR6_23, BR6_24, BR8_31, BR8_32, BR10_39, BR10_40, BR12_47, BR12_48, BR14_55, BR14_56, BR16_63, BR16_64, BR18_71, BR18_72, BR20_79, BR20_80, nCoV-2019_98_RIGHT.

**PCR primer mix A (PPM_A ):** 1.5 µl of each primer (forward and reverse primer) plus 439 µl PCR grade water. = 41 primers (100 pmol/µl) = 4100 pmol in 500 µl water = 8.2 pmol/µl in the PPM. When 2 µl PPM used per 25 µl PCR reaction = 0.66 pmol/µl in reaction (= 660 nM).

**Primers in PPM_A:** nCoV-2019_1_LEFT, AF1_1, AF1_2, AR1_3, AR1_4, AF3_9, AF3_10, AR3_11, AR3_12, AF5_17, AF5_18, AR5_19, AR5_20, AF7_25, AF7_26, AR7_27, AR7_28, AF9_33, AF9_34, AR9_35, AR9_36, AF11_41, AF11_42, AR11_43, AR11_44, AF13_49, AF13_50, AR13_51, AR13_52, AF15_57, AF15_58, AR15_59, AR15_60, AF17_65, AF17_66, AR17_67, AR17_68, AF19_73, AF19_74, AR19_75, AR19_76 .

**PCR primer mix B (PPM_B ):** 1.5 µl of each primer (forward and reverse primer) plus 439 µl PCR grade water = 41 primers (100 pmol/µl) = 4100 pmol in 500 µl water = 8.2 pmol/µl in the PPM. When 2 µl PPM used per 25 µl PCR reaction = 0.66 pmol/µl in reaction (= 660 nM). **Primer Boost:** for primers BF2_5, BF2_6, BR2_7, BR2_8, BF16_62, BF16_61, BR16_63, BR16_64, use 4.5 µl of 100 µM stock

**Primers in PPM_B:** BF2_5, BF2_6, BR2_7, BR2_8, BF4_13, BF4_14, BR4_15, BR4_16, BF6_21, BF6_22, BR6_23, BR6_24, BF8_29, BF8_30, BR8_31, BR8_32, BF10_37, BF10_38, BR10_39, BR10_40, BF12_45, BF12_46, BR12_47, BR12_48, BF14_53, BF14_54, BR14_55, BR14_56, BF16_61, BF16_62, BR16_63, BR16_64, BF18_69, BF18_70, BR18_71, BR18_72, BF20_77, BF20_78, BR20_79, BR20_80, nCoV-2019_98_RIGHT.

**1. Set up Reverse Transcription reactions (two reactions per sample).**

Mix the following components in a 0.2mL 8-strip tube, 1 RT_A and 1 RT-B reaction per sample.

Component Volume (per sample)

**RT_A_Reaction**

**Component Volume**

RPM_A 1 µl

Template RNA 11µl (if using less than 11 µl viral RNA adjust to 11µl with water)
Total Volume 12µl

**RT_B_Reaction**

**Component Volume**

RPM_B 1 µl

Template RNA 11µl (if using less than 11 µl viral RNA adjust to 11µl with water)
Total Volume 12µl

Heat at 65 °C for 5:00 mins, and quickly chill in ice/water bath for at least 1:00 min.


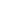


Viral RNA input from a clinical sample should be between Ct 18-35. If Ct is between 12-15, then dilute the sample 100- fold in water, if between 15-18 then dilute 10-fold in water. This will reduce the likelihood of PCR-inhibition.

**Reverse Transcription**

Add the following to the 12 µl annealed template RNA sample:

**Component Volume (1 reaction)**

SuperScript III 5X Buffer 4 µl

100mM DTT 1 µl
RNaseOUT RNase Inhibitor 1 µl

10mM dNTPs mix (10mM each) 1µl

SuperScript III 1 µl

(add 8 µl mix per reaction)

Total Volume 20 μl

**2. Incubate the RT reaction as follows:**

42 °C 50 minutes

70 °C 10 minutes

Hold at 4 °C

**3. Set up Amplicon PCR**

In the Mastermix hood set up the multiplex PCR reactions as follows in 0.2mL 8-strip PCR tubes. Prepare one PPM_A and one PPM_B mix per sample.

**Component 1 reaction 8rxns_ PPM_A_Mix 8rxns_ PPM_B_Mix**

5 x Phusion HF buffer 5µl 40 µl 40 µl

10 mM dNTPs 0.5µl 4 µl 4 µl

Phusion DNA Pol 0.5µl 4 µl 4 µl

Primer PPM_A or PPM_B

(10μM) 2 µl 16 µl 16 µl

Nuclease-free water 12 µl 96 µl 96 µl
Total 20 µl per rxn

Aliquot 20 µl per tube PPM_A_Mix or PPM_B_Mix

In the extraction and sample addition cabinet add **5μl cDNA** to each tube and mix well by pipetting.

**4. Run PCR**

**Step Cycles Temp Time**

Heat Activation 1 98 °C 30sec

Denaturation 35 98 °C 15sec

Annealing 35 63 °C 30sec

Extension 35 72 °C 3 min

Polish 1 72 °C 10 min
Hold 4 °C infinite

In post-PCR cabinet: After PCR, for each sample, pool the two reactions in a 1.5 ml microcentrifuge tube.

**5. Clean up using AMPure XP beads, Agencourt.**

Add an equal volume (1:1) of AMPure XP beads to the sample tube and mix gently by either flicking or pipetting. E.g. add 50 μl AMPure XP bead suspension to a 50 μl reaction.

Pulse centrifuge to collect all liquid at the bottom of the tube.

Incubate for 5 min at room temperature.

Place on magnetic rack and for 2 min

Carefully remove and discard the supernatant, being careful not to touch the bead pellet.

**Ethanol wash 1:** Add 200μl of room-temperature 70%volume ethanol to the pellet.

Carefully remove and discard ethanol, being careful not to touch the bead pellet.

**Repeat Ethanol wash**

Pulse centrifuge to collect all liquid at the bottom of the tube and carefully remove as much residual ethanol as possible using a P10 pipette.

Open lid, allow to dry briefly (2 min)

Resuspend pellet in 30μl Elution Buffer (EB, QIAGEN)

Mix gently by flicking,

Incubate 5 min

Place on magnet and transfer sample (supernatant) to a clean 1.5mL Eppendorf tube ensuring no beads are transferred into this tube.

**6. Quantitate** (A Tapestation 4200 analyzer with the D5000 assay kit as well as the Qubit fluorometer were used.)

**7. Add Barcodes (Steps 7 onward derived from the Oxford Nanopore Technologies protocol (1)).**

Set up the following reaction for each sample:

**Component Volume**

DNA amplicons 5 µl*
Nuclease-free water 7.5 µl
Ultra II End Prep Reaction Buffer 1.75 µl

Ultra II End Prep Enzyme Mix 0.75 µl

Total Volume 15 µl

*Volumes usually varies based on the DNA concentration. For the 1500bp amplicons and pool size of appx 8 we have input 50ng and for a pool size of 12 we have input 20ng

Incubate at room temp (20°C) for 20 min

Incubate at 65 °C for 5:00 min

Incubate on ice for 1 min

E7645S/L NEBNext Quick Ligation Module

**Component Volume**

End repaired sample 15µl

Barcode vol 2.5 µl

Ultra II Ligation Master Mix 17.5 µl

Ligation Enhancer 0.5 µl

Water 1.5 µl

final volume 35.5 µl

Incubate in a thermocycler for 15 minutes at 20°C with heated lid set to 30°C (or off).

Incubate at 70 °C for 10 min

Incubate on ice for 1 min

The 70°C incubation is to inactivate the DNA ligase to prevent barcode cross-ligation when reactions are pooled in the next step.

Pool all barcoded fragments together into a new 1.5 ml Eppendorf tube.

**8. Clean up on AMPure XP beads, Agencourt.**

Add an equal volume (1:1) of SPRI beads to the sample tube and mix gently by either flicking or pipetting. E.g. add 50 μl SPRI beads to a 50 μl reaction.

Pulse centrifuge to collect all liquid at the bottom of the tube.

Incubate for 5 min at room temperature.

Place on magnetic rack and for 5 min

Carefully remove and discard the supernatant, being careful not to touch the bead pellet.

**Ethanol wash 1:** Add 200μl of room-temperature 70%volume ethanol to the pellet.

Carefully remove and discard ethanol, being careful not to touch the bead pellet.

**Repeat Ethanol wash**

Pulse centrifuge to collect all liquid at the bottom of the tube and carefully remove as much residual ethanol as possible using a P10 pipette.

Open lid, allow to dry briefly (2 min)

Resuspend pellet in 30μl Elution Buffer (EB, QIAGEN)

Mix gently by flicking

Incubate 5 min

Place on magnet and transfer sample (supernatant) to a clean 1.5mL Eppendorf tube ensuring no beads are transferred into this tube.

**9. Quantitate as above.**

**10. Add AMII adapters.**

AMII adapters ligation reaction:

**Component Volume**

Barcoded amplicon pools 30 µl
NEBNext (E60562)

Quick Ligation Reaction Buffer (5X) 10 µl

AMII adapter mix 5 µl
Quick T4 DNA Ligase 5 µl
Total 50 µl

The input of barcoded amplicon pools will depend on the number of barcoded pools and should be between 40 ng (8 barcodes) and 160 ng (24 barcodes).

Incubate at room temperature for 15:00 min

**11. Clean up on AMPure XP beads, Agencourt.**

**USE SFB instead of Ethanol for two washes ***CRUCIAL,** Ethanol strips off motor protein from adapter. You don't want to do this!

Clean up on AMPure XP beads, Agencourt

Add an equal volume (1:1) of SPRI beads to the sample tube and mix gently by either flicking or pipetting. E.g. add 50 μl SPRI beads to a 50 μl reaction.

Pulse centrifuge to collect all liquid at the bottom of the tube.

Incubate for 5 min at room temperature.

Place on magnetic rack and for 2 min

Carefully remove and discard the supernatant, being careful not to touch the bead pellet.

**SFB washes:** Add 200μl of room-temperature **SFB** to the pellet.

resuspend

collect on magnet

Carefully remove and discard **SFB**  being careful not to touch the bead pellet.

**Repeat SFB wash**

Pulse centrifuge to collect all liquid at the bottom of the tube and carefully remove as much residual **SFB** as possible using a P10 pipette.

**Resuspend pellet in 15 µl Elution Buffer (EB, QIAGEN)**

Mix gently by flicking,

**Incubate 5 min**

Place on magnet and transfer sample (supernatant) to a clean 1.5mL Eppendorf tube ensuring no beads are transferred into this tube.

**12. Quantitate.**

**13. Proceed to MinION sequencing.**

**3. Supplementary material Table 1. Entebbe Primers**

| **Amplicon_no** | **Primer_id** | **Sequence^1^** | **Position^2^** |
| --- | --- | --- | --- |
| 1 | AF1_1 | GTCCGGGTGTGACCGAAAG | 242 |
| 1 | AF1_2 | CCTTGTCCCTGGTTTCAACGA | 274 |
| 1 | AR1_3 | TGCGGGAGAAAATTGATCGTACA | 1894 |
| 1 | AR1_4 | GCACAGAATTTTGAGCAGTTTCA | 1921 |
| 2 | BF2_5 | ACGTGCTAGCGCTAACATAGG | 1540 |
| 2 | BF2_6 | GTTGGAGAAGGTTCCGAAGGT | 1580 |
| 2 | BR2_7 | TAGCCTTATTTAAGGCTCCTGCAA | 3477 |
| 2 | BR2_8 | TGCAACACCTCCTCCATGTTTAA | 3459 |
| 3 | AF3_9 | ACAAACTGTTGGTCAACAAGACG | 3220 |
| 3 | AF3_10 | CAACAAGACGGCAGTGAGGA | 3233 |
| 3 | AR3_11 | TTGTGTAGATTGTCCAGAATAGGACC | 4806 |
| 3 | AR3_12 | TCCATATGTCATTGACATGTCCACA | 5014 |
| 4 | BF4_13 | TTTGGAAGAAGCTGCTCGGTATAT | 4639 |
| 4 | BF4_14 | GAAACCATCTCACTTGCTGGTTC | 4772 |
| 4 | BR4_15 | GGTTTTAGATCTTCGCAGGCAAG | 6380 |
| 4 | BR4_16 | CCATTAGATCTGTGTGGCCAA | 6534 |
| 5 | AF5_17 | CACACCCTCTTTTAAGAAAGGAGCTA | 6190 |
| 5 | AF5_18 | TTGTTTGGCATGTTAACAATGCAAC | 6234 |
| 5 | AR5_19 | CAGACGCTGATTTTGCAGATGA | 7919 |
| 5 | AR5_20 | GCACTATCACCAACATCAGACAC | 7994 |
| 6 | BF6_21 | AGGCTTTTGCAAACTACACAATTG | 7591 |
| 6 | BF6_22 | TATGCTAATGGAGGTAAAGGCTTTTG | 7574 |
| 6 | BR6_23 | CGATAGCTACAATACCACCAGCT | 9409 |
| 6 | BR6_24 | CAATACCACCAGCTACTATAGATGCT | 9397 |
| 7 | AF7_25 | AAGCTGGTGTTTGTGTATCTACTAGT | 9243 |
| 7 | AF7_26 | ACCTACCTTGAAGGTTCTGTTAGAG | 9164 |
| 7 | AR7_27 | CACTACCCAATATGGTACGTCCA | 10885 |
| 7 | AR7_28 | TGAGTAACAACCAGTGGTGTGT | 11000 |
| 8 | BF8_29 | CTGGAGTTCATGCTGGCACA | 10560 |
| 8 | BF8_30 | GTTTTAGCTTGGTTGTACGCTG | 10664 |
| 8 | BR8_31 | TTTGCCCTCTTGTCCTCAGATCTA | 12313 |
| 8 | BR8_32 | TGGTATGACAACCATTAGTTTGGCT | 12466 |
| 9 | AF9_33 | CTCAAGAAGCTTATGAGCAGGCT | 12144 |
| 9 | AF9_34 | CAAGCTATAGCCTCAGAGTTTAGTTC | 12089 |
| 9 | AR9_35 | ACGTTGACGTGATATATGTGGTACC | 13770 |
| 9 | AR9_36 | ACGAGGTCTGCCATTGTGTATT | 13802 |
| 10 | BF10_37 | GCGGTGTAAGTGCAGCC | 13475 |
| 10 | BF10_38 | TCGCTTCCAAGAAAAGGACGAA | 13602 |
| 10 | BR10_39 | CTCAATACTTGAGCACACTCATTAGC | 15406 |
| 10 | BR10_40 | GTGACAAGCTACAACACGTTGT | 15367 |
| 11 | AF11_41 | ATTCTATGGTGGTTGGCACAACA | 15219 |
| 11 | AF11_42 | CAATAGCCGCCACTAGAGGAG | 15173 |
| 11 | AR11_43 | TAGTGTAGGTGCACTTAATGGCATT | 16932 |
| 11 | AR11_44 | GGTAAACAACAGCATCACCATAGTC | 16846 |
| 12 | BF12_45 | AACATGTGACTGGACAAATGCTG | 16566 |
| 12 | BF12_46 | ACTGACTTTAATGCAATTGCAACATG | 16546 |
| 12 | BR12_47 | TACAGCAACTAGGTTAACACCTGTAG | 18374 |
| 12 | BR12_48 | CACCCCTCGACATCGAAGC | 18302 |
| 13 | AF13_49 | GTGGCAACTTTACAAGCTGAAAATG | 18025 |
| 13 | AF13_50 | GACATACCTGGCATACCTAAGGAC | 18160 |
| 13 | AR13_51 | GTTTAATGTTGCGCTTAGCCCAA | 19791 |
| 13 | AR13_52 | GTAGTCCCAGATCACAGTATTAGCAG | 19859 |
| 14 | BF14_53 | CGTGTATAACACGTTGCAATTTAGGT | 19454 |
| 14 | BF14_54 | ACTTTGATGGACAACAGGGTGAA | 19661 |
| 14 | BR14_55 | CGCGTGGTTTGCCAAGATAATTA | 21285 |
| 14 | BR14_56 | CATAACCATCTATTTGTTCGCGTGG | 21301 |
| 15 | AF15_57 | AGCTCATGGGACACTTCGC | 21203 |
| 15 | AF15_58 | TTGGAGGTTCCGTGGCTATAAAG | 21146 |
| 15 | AR15_59 | CGATTTGTCTGACTTCATCACCTC | 22770 |
| 15 | AR15_60 | CTACCGGCCTGATAGATTTCAG | 22971 |
| 16 | BF16_61 | TGCATCTGTTTATGCTTGGAACAG | 22603 |
| 16 | BF16_62 | CTCTCTCAGAAACAAAGTGTACGTTG | 22446 |
| 16 | BR16_63 | CACTTGCTGTGGAAGAAAGTGA | 24371 |
| 16 | BR16_64 | GTTGACCACATCTTGAAGTTTTCCAA | 24396 |
| 17 | AF17_65 | GGTGATTGCCTTGGTGATATTGC | 24074 |
| 17 | AF17_66 | CTGTTTTGCCACCTTTGCTCA | 24138 |
| 17 | AR17_67 | CCAGCAAAGAAAATAGTTGGCATC | 25816 |
| 17 | AR17_68 | CGTAACAATTAGTATGCCAGCAAAGA | 25830 |
| 18 | BF18_69 | TCCCTTTCGGATGGCTTATTGTT | 25514 |
| 18 | BF18_70 | TGAAATCAAGGATGCTACTCCTTCAG | 25446 |
| 18 | BR18_71 | GTTGTACCTCTAACACACTCTTGGTA | 27451 |
| 18 | BR18_72 | CTCACAAGTAGCGAGTGTTATCAG | 27418 |
| 19 | AF19_73 | ACGCTTTCTTATTACAAATTGGGAGC | 27045 |
| 19 | AF19_74 | CGCTGTGACATCAAGGACCT | 26994 |
| 19 | AR19_75 | TGGCAATGTTGTTCCTTGAGGAA | 28755 |
| 19 | AR19_76 | CAGCCATTCTAGCAGGAGAAGTT | 28885 |
| 20 | BF20_77 | TAGAGTATCATGACGTTCGTGTTGTT | 28219 |
| 20 | BF20_78 | ACCCCGCATTACGTTTGGT | 28309 |
| 20 | BR20_79 | TGGCTCTTTCAAGTCCTCCCT | 29700 |
| 20 | BR20_80 | GCTCTTCCATATAGGCAGCTCTC | 29779 |
|  | nCoV-2019_1_LEFT^3^ | ACCAACCAACTTTCGATCTCTTGT | 31 |
|  | nCoV-2019_98_RIGHT^3^ | TTCTCCTAAGAAGCTATTAAAATCACATGG | 29866 |

**Footnotes** 1. Sequence listed 5' to 3'.

2. Position in SARS-CoV-2 reference genome GenBank NC_045512

3. From original ARTIC primer set V.1 (2).

1. Oxford Nanopore Technologies. Nanopore Protocol PCR tiling of COVID-19 virus. 2020 [cited 2021 Jan 24]; Available from: https://community.nanoporetech.com/protocols/pcr-tiling-ncov/v/PTC_9096_v109_revH_06Feb2020

2. Josh Quick. nCoV-2019 sequencing protocol. 2020; Available from: https://www.protocols.io/view/ncov-2019-sequencing-protocol-bbmuik6w

3. Hsin-Ru Chang, Jay Crespo, Kathy Lee, Kathleen McGall, Susan MacWhorter, Paul Russell, Jeff Schatz, Donna Seid, Criss Walworth, Joe Yu. TaqMan® Assays Shipped at Ambient Temperature Reduce Environmental Impact and Retain Their Quality and Stability. [cited 2021 Jan 24]; Available from: https://assets.thermofisher.com/TFS-Assets/LSG/brochures/cms_081489.pdfhttps://assets.thermofisher.com/TFS-Assets/LSG/brochures/cms_081489.pdf
